# Cooperation of Conical and Polyunsaturated Lipids to Regulate Initiation and Processing of Membrane Fusion

**DOI:** 10.3389/fmolb.2021.763115

**Published:** 2021-10-21

**Authors:** Claire François-Martin, Amélie Bacle, James E. Rothman, Patrick F. J. Fuchs, Frédéric Pincet

**Affiliations:** ^1^ Laboratoire de Physique de l’Ecole Normale Supérieure, ENS, CNRS, Université PSL, Sorbonne Université, Université de Paris, Paris, France; ^2^ Laboratoire Coopératif “Lipotoxicity and Channelopathies—ConicMeds”, Université de Poitiers, Poitiers, France; ^3^ Department of Cell Biology, School of Medicine, Yale University, New Haven, CT, United States; ^4^ Nanobiology Institute, Yale School of Medicine, West Haven, CT, United States; ^5^ Laboratoire des Biomolécules (LBM), CNRS, Ecole Normale Supérieure, Sorbonne Université, PSL Research University, Paris, France; ^6^ UFR Sciences Du Vivant, Université de Paris, Paris, France

**Keywords:** activation energy, fusion, lipid shape, fusion kinetics, molecular dynamics simulations

## Abstract

The shape of lipids has long been suspected to be a critical determinant for the control of membrane fusion. To experimentally test this assertion, we used conical and malleable lipids and measured their influence on the fusion kinetics. We found that, as previously suspected, both types of lipids accelerate fusion. However, the implicated molecular mechanisms are strikingly different. Malleable lipids, with their ability to change shape with low energy cost, favor fusion by decreasing the overall activation energy. On the other hand, conical lipids, with their small polar head relative to the area occupied by the hydrophobic chains, tend to make fusion less energetically advantageous because they tend to migrate towards the most favorable lipid leaflet, hindering fusion pore opening. They could however facilitate fusion by generating hydrophobic defects on the membranes; this is suggested by the similar trend observed between the experimental rate of fusion nucleation and the surface occupied by hydrophobic defects obtained by molecular simulations. The synergy of dual-process, activation energy and nucleation kinetics, could facilitate membrane fusion regulation *in vivo*.

## Introduction

**FIGURE 1:**
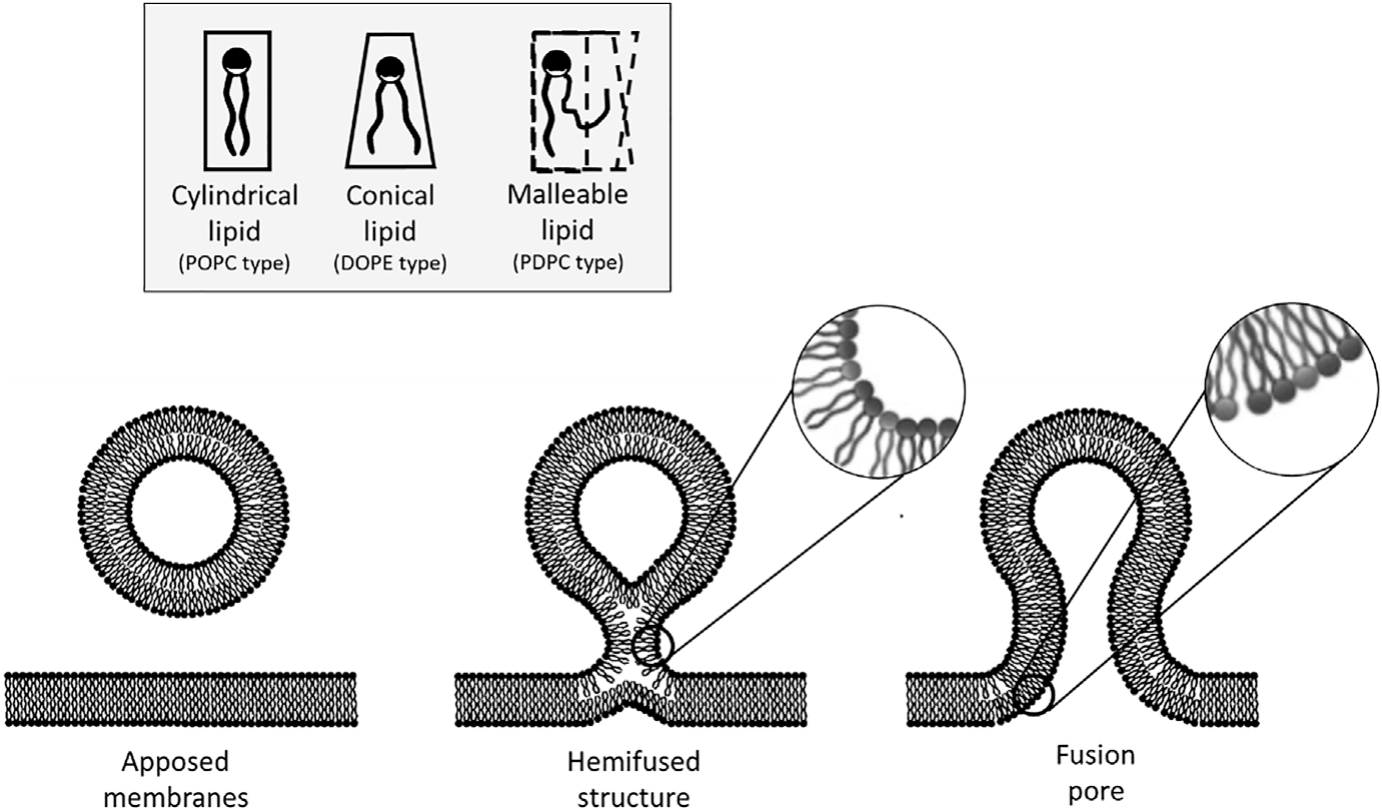
Steps of the membrane fusion process and considerations on the energy impact of lipid geometry. The fusion process starts by the formation of an hemifused-like structure (only the outer leaflets have merged). This highly curved structure spatially constrains the hydrophilic heads of the lipids that are situated on the outer leaflet. Due to their spontaneous curvature, it is more favorable energetically for conical lipids to adopt this configuration than cylindrical lipids. Fusion is completed by the opening of a pore. At this step, the inner leaflets of the bilayer are now curved, and in this case, it is less favorable for conical lipids to adopt this configuration as their hydrophobic tails are now more accessible to water. On the other hand, malleable lipids could favor any bent configuration as they can adapt their geometry to that of the membrane without significant energy cost.

Membrane fusion is one of the means used by cells, organelles and lipid-bound objects to interact and transmit information (Martens and McMahon, 2008). Communication takes place as the contents of the newly fused compartments freely mix or react after the lipids leaflets of two compartments have coalesced to form a unique and continuous membrane. Erratic membrane fusion is prevented by the energy barriers that ought to be overcome on the pathway to fusion (Kuzmin et al., 2001). It is now quite commonly assumed that fusion proceeds in steps: first, through the approach and binding of the apposed membranes, then by the formation of an hemifused-like structure in which only the outer monolayers have fused, and finally by the completion of fusion through the merging of the inner monolayers thereby forming a fusion pore (Figure 1). *In vivo*, the necessary energy can be brought by proteins. For instance, in the case of intracellular vesicular transport and exocytosis, SNARE proteins that are present on two apposing membranes spontaneously assemble into an energetically favorable coiled-coil structure (Söllner et al., 1993; Weber et al., 1998; Li et al., 2007). The energy released during the formation of this protein complex is harvested to overcome the energy barriers separating the different intermediates on the fusion pathway (Wiederhold and Fasshauer, 2009; Risselada and Grubmüller, 2012; Ryham et al., 2016).

At the molecular level, many pathways can be envisioned for the fusion process which make the resulting energy landscape difficult to accurately apprehend. A simplified view in which the molecular details are averaged is probably the best approach to provide a good description of the fusion kinetics. We recently showed experimentally on membranes made of a single lipid type (DOPC or POPC, see Material and Methods for the definition of lipid acronyms) that the fusion kinetics are well-described by an Arrhenius law, *i.e.* that the energy landscape for fusion can be approximated by a single energy barrier over a range of physiologically relevant temperatures (27°C–47°C) (François-Martin et al., 2017). The corresponding activation energy, about 30 k_B_T, is high enough to prevent spontaneous fusion but sufficiently low to be tunable by proteins for rapid fusion.

Here we address the question whether the lipid composition of the membrane can affect the energy landscape for fusion. We focus specifically on the role of lipid geometry and flexibility/malleability. Using bulk fusion assay, we show that both conical shape lipids and malleable lipids accelerate fusion kinetics. However, they act on the fusion process through different mechanisms. While flexible lipids reduce the activation energy for fusion, conical lipids increase the nucleation rate of fusion, *i.e.* the prefactor in the Arrhenius law, by increasing the density of hydrophobic defects exposed to the outer medium. This led us to propose that both processes for changing fusion kinetics could actually be modulated *in vivo* to facilitate fusion regulation. To test this hypothesis, we mimicked the fusion of a synaptic vesicle and a typical plasma membrane. We find that, in this case, fusion displays both a low activation energy (about 23 k_B_T) and a low initiation constant (about 10^6^ min^−1^ compared to 10^12^ min^−1^ for DOPC and 10^8^ min^−1^ for POPC membranes). This feature could enable a tight control of the pre-synaptic fusion and allow neurotransmission’s fine regulation. Indeed, having a low activation energy facilitates vesicle fusion. Yet, the low initiation rate renders spontaneous fusion highly improbable, the latter potentially only being nucleated after membrane destabilization by SNARE proteins.

## Results and Discussion

### Conical and Malleable Lipids Accelerate Fusion Kinetics

The ability of lipids to exhibit favored shapes has long been suspected to play a role in controlling membrane fusion by promoting or inhibiting some steps of the fusion process. For instance, a mismatch between a small polar head and larger hydrophobic chains of a lipid provides it with a conical shape and drives a local spontaneous curvature of the membrane. This conical shape can favor or hinder fusion according to the correlation between the lipids’ spontaneous curvature and the curvature of the different intermediate structures (Chernomordik and Zimmerberg, 1995; Chernomordik, 1996). Previous studies suggest that conical lipids, *e.g.* lipids having a small polar head compared to their hydrophobic tails and thus having a natural negative curvature, favor fusion (Yèagle, 1989). It was also suggested that malleable lipids, *i.e.* lipids that can adopt various shapes with limited energy cost, may also favor fusion (Pinot et al., 2014). Our goal is to test this hypothesis that lipid shape and malleability can play a critical role in the fusion process. Hence, we focused on these two features, shape and malleability of the lipid, and picked two lipids that are known to be either conical (DOPE) or malleable (a polyunsaturated lipid, PDPC).

**FIGURE 2:**
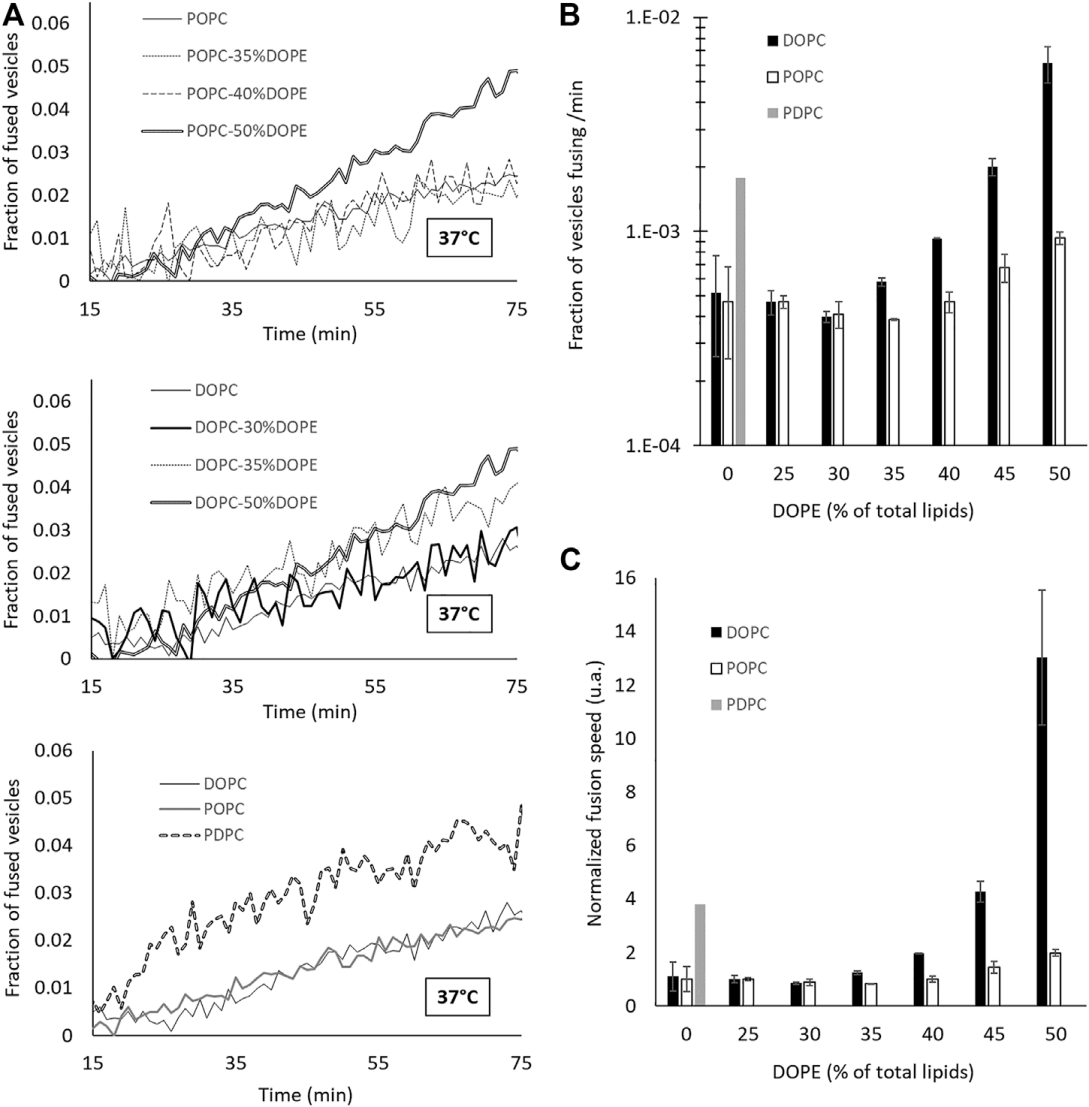
*Adding DOPE in a phosphatidylcholine (PC) membrane or increasing the degree of unsaturation of the PC tails leads to an increase of the fusion speed.* ***(A)*** *Dequenching curves at 37 °C for different lipid compositions. For the sake of clarity, not all the intermediate curves are represented. However, all the data are presented in* ***(B,C)*** *.* ***(B–C)*** *Speed of fusion (in fraction of the total vesicles which undergo a fusion event in 1 minute) for different compositions on a semi-log scale* ***(B)*** *and on a linear scale with values normalized by that of pure POPC fusion speed* ***(C)*** *.*

To measure fusion kinetics, we used a well-established protocol (Struck et al., 1981) which consists in following a change in Förster resonance energy transfer (FRET) signal, that can be linked to the number of fusion events. In brief, two sets of vesicles were mixed together—one containing N-(7-Nitrobenz-2-Oxa-1,3-Diazol-4-yl (NBD) and Lissamine Rhodamine (Rh) lipids at a concentration where NBD is quenched by the presence of Rh, and one containing no dyes. The fusion between a vesicle of each population leads to a dilution of the dyes and hence to an increase in the NBD signal (Weber et al., 1998; François-Martin et al., 2017; François-Martin and Pincet, 2017). We made vesicles having POPC:DOPE and DOPC:DOPE mixtures with 0, 25, 30, 35, 40, 45 and 50% DOPE, and vesicles made of PDPC. All experiments were performed with 18 mM lipids in an HEPES 25 mM—KCl 100 mM—pH 7.4 buffer. The NBD dequenching, monitored in time at 37°C, shows that the kinetics of fusion is accelerated when DOPE is added beyond 35% and with PDPC (Figure 2A). To quantify this increase, we plotted the initial slope and translated it in terms of fraction of vesicles fusing per minute (Figure 2B). Figure 2C represents the ratio of increase of the fusion speed compared to that of pure POPC membranes. Both DOPE and PDPC are able to enhance the rate of fusion by one order of magnitude compared to POPC alone. This result confirms that, in agreement with the common wisdom, conical and malleable lipids favor membrane fusion. However, we will show in the next sections that this facilitation of membrane fusion is not necessarily attributed to the correct underlying causes.

### Thermodynamic Origin of the Enhanced Fusion

Our goal is now to explain how these lipids can favor fusion. Before detailing the experimental results, it is necessary to provide a description of membrane fusion at the molecular level and introduce the notions of activation energy and nucleation rates for the fusion process. Let’s consider the case of two non-adhering 80 nm vesicles that collide. Fusion must occur during the time their membranes are in direct interaction, *i.e.* typically are less than 1–10 nm apart (Aeffner et al., 2012) which corresponds to a few characteristics decay length of the short-range hydration and protrusion repulsions (Rand and Parsegian, 1989). Depending on their incidence angle, this corresponds to a travel distance of about 2–20 nm and, according to the Stokes-Einstein relation, a travel time between 60 ns and 6 µs. The fusion process must be nucleated during these 1 µs. Hence, at the molecular level, the lipids must reorganize so that the membranes reach a quasi-irreversible state leading to fusion. In the membrane plane, lipids exchange position on the scale of 100 ns. Similarly, the orthogonal movement of the lipid (protrusions) also takes place on 100 ns timescale (Tahir et al., 2016). Thus, while the vesicles are interacting during a collision event, lipids can reorganize but with only a limited number of accessible conformations. This shows that, when a fusion event occurs, membrane remodeling at the molecular level will be very dependent on the local lipid conformation at the time of collision. This dependency on the initial state explains why two membrane fusion events will follow a different pathway at the molecular level and makes detailed modeling extremely complex. To circumvent this issue, we chose not to focus on a single fusion event but, instead, consider an average fusion pathway over many events disregarding the specific lipid arrangement of every single event. On the example of vesicles made of standard neutral phospholipids, POPC or DOPC, we have previously monitored trillions of fusion events at various temperatures in the vicinity of 37°C and showed that the speed of fusion expressed in rounds of fusion per second per molar follows an Arrhenius-like law: ν = ν_0_ exp (−E_A_/k_B_T) (François-Martin et al., 2017). This result justifies the use of a simplified model in which the detailed molecular organization is not defined but can be mathematically described by a single global energy barrier. In this context fusion kinetics are simply described by two parameters: E_A_ that appears as the apparent activation energy of the fusion process and ν_0_ which is the nucleation frequency, *i.e.* the frequency at which the lipids in the interacting regions of the vesicles adopt a conformation susceptible to lead to fusion. Because the model is simplified, these values are only valid at 37°C. For instance, ν_0_, which should not depend on the temperature, will actually vary with the temperature, though it can be considered constant over the range used here (27–47°C) because of the exponential variation of the speed of fusion in the experiments. The variations of ν_0_ are due to the changes in lipid organization and dynamics with temperature.

**FIGURE 3:**
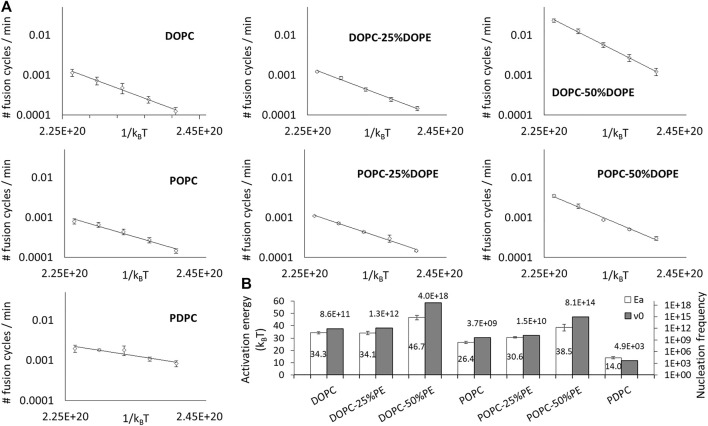
Lipid composition impacts the activation energy and the nucleation rate of fusion. **(A)** The variation of the initial spontaneous fusion speeds are plotted against the temperature (from 27 to 47°C) for DOPC, DOPC-25%DOPE, DOPC-50%DOPE, POPC, POPC-25%DOPE, POPC-50% DOPE, and PDPC. One fusion cycle corresponds to 100% of the vesicles of the sample which have undergone fusion. For the sake of clarity, the averages of the fusion speeds of the independent experiments are represented (error bars are standard errors on the mean) even though the activation energies values in **(B)** were deduced from independent fits. **(B)** The activation energy values and nucleation frequencies are represented for the different lipid compositions. Activation energies (empty bars) are determined through independent fits of 3–9 different experiments and the error bars correspond to the standard errors on the mean. Nucleation frequencies (filled bars) are determined from the average fit.

According to this paradigm, the change in fusion kinetics with conical and malleable lipids can only be attributed to the activation energy and/or the nucleation frequency. To test this hypothesis, we performed the same fusion measurements as before, but over a range of temperatures running from 27°C to 47°C with a 5°C step for DOPC, POPC, DOPC:DOPE (75:25), POPC:DOPE (75:25), DOPC:DOPE (50:50), POPC:DOPE (50:50) and PDPC. We found that all the corresponding fusion speeds displayed an Arrhenius law behavior (Figure 3A). Both the corresponding energy barriers and nucleation frequencies (Figure 3B) display large variations with the shape and malleability of lipids.

The only result that can be readily explained in this set of data is the decreased activation energy with PDPC. Indeed, because polyunsaturated are known to be very malleable, *i.e.* they can change shape with little energy cost, it is intuitively clear that the molecular remodeling required during the fusion process will require much fewer thermal fluctuations to be spontaneously achieved than with non-malleable lipids.

Uncovering the molecular origin of the increased of activation energy with conical lipids and changes in nucleation rate is not straight-forward and requires additional experiments.

### Lipids With Negative Spontaneous Curvature Are Preferentially Located On The Inner Leaflet Of Liposomes Which Increases The Activation Energy For Fusion

The current hypothesis is that the geometry of conical lipids could promote fusion by lowering the energy of hemifusion (Siegel, 1993; Haque et al., 2001; Kozlovsky et al., 2002). However, hemifusion is not necessarily the limiting step in the fusion process. Notably, the presence of conical lipids with negative curvatures could also hinder the merging of the inner leaflets, *i.e.* the opening of the fusion pore. Our results show that adding DOPE monotonically increases the fusion activation energy (Figure 3B).

Intuitively, it makes sense that, when distributed between the membrane leaflets of a liposome, lipids favoring negative curvature will spontaneously tend to insert in the negatively curved inner leaflet rather that in the positively curved outer leaflet. This effect should be more significant as the curvature of the membrane is increased. Thus, conical shape lipids could stabilize membranes and, against the common wisdom, actually inhibit fusion by increasing the overall activation energy. This uneven distribution of the lipids can be estimated by a simple model assuming a binary system in which the spontaneous curvature of the other lipid is zero, which is a good approximation for DOPC or POPC, and ignoring the area difference between the inner and outer leaflet. In this model, the energy of the conical lipid in the inner leaflet and the outer leaflet are respectively: $e_{i}=2\kappa a{\left(c_{0}+\frac{1}{R}\right)}^{2}$ and $e_{o}=2\kappa a{\left(c_{0}-\frac{1}{R}\right)}^{2}$ , where κ is the bending modulus, $c_{0}$ the spontaneous curvature of the conical lipid and R the local mean radius of the membrane. Hence the energy change when a lipid flips from the inner to the outer leaflet is $\text{Δ}e=e_{o}-e_{i}=-\frac{8\kappa ac_{0}}{R}$ . As expected, this change is positive for a conical lipid with a negative spontaneous curvature, indicating it will preferably insert in the inner leaflet. Hence, r , the ratio between the number of conical lipids in the outer leaflet and in the inner leaflet is:

r=exp(−ΔekBT) (1)

the fraction of conical lipid in the outer leaflet is:

$$f_{o}=\frac{2fr}{1+r}$$ (2)

and the fraction of conical lipid in the inner leaflet is:

$$f_{i}=\frac{2f}{1+r}$$ (2)

where f is the input fraction of conical lipid.

Note that, in this simple model, r does not depend on f . For DOPE, $c_{0}$ is of the order of −0.4 nm^−1^ (Kollmitzer et al., 2013), *i.e.* a spontaneous curvature of 2.5 nm, κ is about 20 $k_{B}T$ , and a∼ 0.65 nm^2^. The typical radius of the liposomes we used was 40 nm (François-Martin et al., 2017). The distribution of DOPE predicted by this model in our experiments is indicated in Table 1.

**TABLE 1:** DOPE is preferentially situated on the inner leaflet of vesicles. $f_{o}$ corresponds to the fraction of the outer leaflet that is occupied by PE lipids whereas $f_{i}$ corresponds to the fraction of the inner leaflet that is occupied by PE lipids. The theoretical values are obtained thanks to curvature energy calculations. The experimental values are obtained thanks to the comparison of the fluorescence intensities of a sample after external PE labeling followed by total PE labeling. The errors correspond to the standard error on the mean of the different experiments. The ratio between the PE occupation of the outer leaflet compared to that of the inner leaflet confirms that there is an enrichment of PE on the inner leaflet.

DOPE input fraction ( f )	DOPE fraction in the inner leaflet ( $f_{i}$ )		DOPE fraction in the outer leaflet ( $f_{o}$ )		Ratio between the outer and inner leaflets ( r )	
	Model	Experiment	Model	Experiment	Model	Experiment
0.25	0.37	0.42 ± 0.01	0.13	0.10 ± 0.01	0.35	0.24 ± 0.04
0.5	0.74	0.84 ± 0.02	0.26	0.27 ± 0.02	0.35	0.33 ± 0.03

To check the validity of the predictions of this model, we directly measured the distribution of DOPE in our DOPC liposomes by sequentially labelling the external DOPE and then the DOPE situated on the internal leaflets of our vesicles. In brief, external DOPE was first labeled by incubating the vesicles with Alexa 647-NHS. The unbound dyes were then removed by dialysis. The quantification of the labeled DOPE was done by measuring Alexa’s fluorescence intensity in bulk after membrane solubilization. The samples were then labeled again by Alexa 647-NHS which could now bind to the newly reachable internal DOPE. After a new dialysis to get rid of the unbound dyes and a re-solubilization, the fluorescence was read again thus allowing to quantify the total amount of DOPE in the solution. The ratio of these two intensities gives the fraction of the DOPE that is present on the external leaflets. Accounting for the total fraction of DOPE in the respective samples, one can determine which fraction of both monolayers are occupied by PE lipids. The results are displayed in Table 1 next to the theoretical predictions.

The results, in strikingly good agreement with the predictions, confirm that the majority of the DOPE is located in the inner leaflet of the membrane. Hence, even though conical lipids with a negative spontaneous curvature are likely to favor hemifusion, they tend to migrate towards the inner leaflet at equilibrium where they are unfavorable to fusion because they hinder pore opening. This uneven distribution of conical lipids explains why the activation energy of fusion increases with the DOPE fraction.

In addition, this observation may also explain the subtle differences between DOPC and POPC. Even though both lipids are globally cylindrical, DOPC is slightly more conical with a spontaneous curvature of −0.091 ± 0.008 nm^−1^, compared to −0.022 ± 0.01 nm^−1^ for POPC (Kollmitzer et al., 2013). This could potentially explain the different effect of DOPE in DOPC versus POPC membranes. Indeed, the difference of geometry is slightly less important between DOPC and DOPE than between POPC and DOPE so the tendency of DOPE to move in the internal leaflet could be less pronounced in DOPC membranes. This is totally in line with the fact that the addition of 50% of DOPE in DOPC (respectively POPC) membranes leads to an increase of the activation energy of 36% (respectively 46%).

### The Nucleation Rate is Correlated With Membrane Surface Hydrophobicity

**FIGURE 4:**
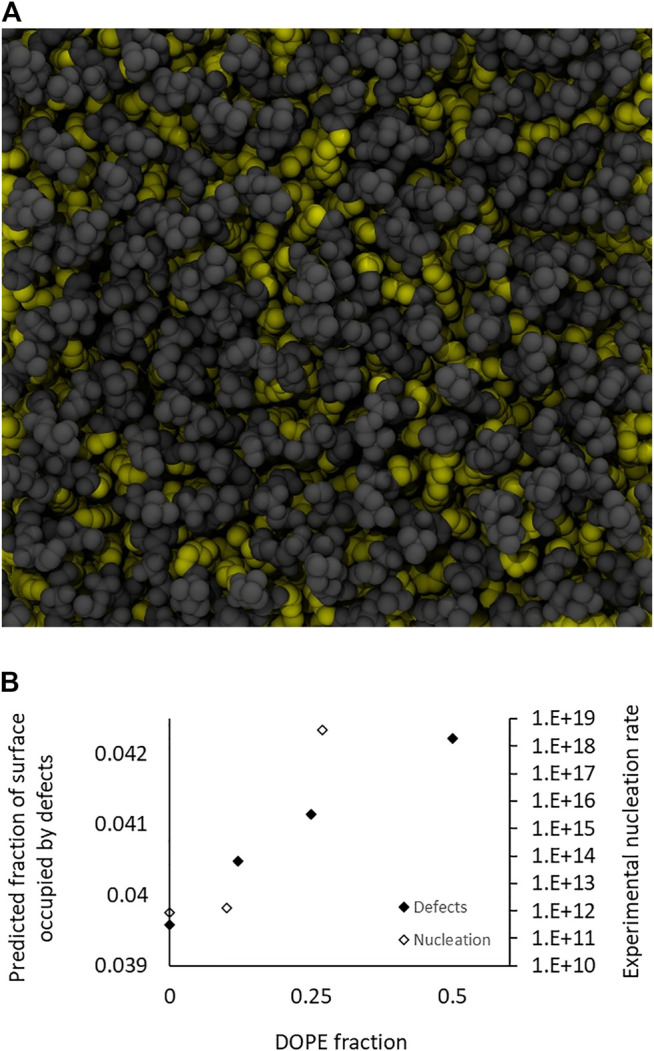
Simulations of defects for 0, 12.5, 25, and 50% DOPE. **(A)** Top view of a simulation snapshot from the DOPC:DOPE (50:50 mol:mol) system. Hydrophobic atoms (starting from the C2 till the end methyl of either sn-1 or sn-2) are represented in yellow van der Waals spheres, polar heads are in gray. The visible yellow atoms represent hydrophobic defects. **(B)** Evolution of the surface fraction occupied by defects in function of the fraction of DOPE lipids. It should be noted that these values may underestimate the actual values in our experiments, where the bilayers are actually curved, therefore increasing the inadequation between the geometry of the external DOPE lipids and that of the membrane. The experimentally measured nucleation rates (from Figure 3B) for total fractions of DOPE of 0, 25, and 50%, corresponding to DOPE fractions on the outer leaflet of respectively 0, 10, and 27% (from Table 1) are also presented and qualitatively exhibit similar variation.

Understanding the variation of the nucleation rate requires to focus on the outer leaflet where the first contact between the two membranes occurs. A striking result is that the nucleation rate monotonically increases with the concentration of lipid favoring negative spontaneous curvatures. Since the nucleation rate is likely due to the local lipid organization, we conducted molecular dynamics simulations of DOPC bilayers with various fractions of DOPE lipids. Following Leikin’s insight that curved membranes composed of cylindrical lipids display many defects (Leikin et al., 1987), conical lipids could, in flat bilayers, expose more of their hydrophobic tails to the aqueous solution than would cylindrical lipids. Intuitively, one could sense that these “hydrophobic defects”, that are not favorable energy-wise, could be preferential zones to nucleate fusion and thus promote fusion. In our numerical simulations, we identified hydrophobic defects, also known as packing defects (Vamparys et al., 2013; Gautier et al., 2018), as areas where the first atoms of the lipids viewed perpendicularly from the surface are in the alkyl chains (yellow atoms in Figure 4A). We then compared the fraction of the total surface area occupied by defects in membranes containing increasing fractions of DOPE lipids (from 0 to 50%) and the results, presented in Figure 4B, show that the surface occupied by defects increases with the fraction of DOPE lipids in the flat bilayers. This is qualitatively in line with our hypothesis that defects could favor fusion by allowing its nucleation. It should be noted that since membranes containing 50% of DOPE (resp. 25% DOPE) actually exhibit outer leaflet that present only 27% (resp 10%) of DOPE, the surface fraction occupied by defects in membranes with input fractions of 25% (resp 12%) are of most interest.

Explaining quantitatively the extremely large variations of the nucleation rates over seven orders of magnitude (10^7^) between the various cases we tested requires a better understanding of the implications of an increased number of hydrophobic defects. It is well established that a lipid exposing more hydrophobic areas (*i.e.* hydrophobic defects) to the solvent, such as DOPE over DOPC, will bind less water molecules and will also tend to hide closer to the hydrophobic. As a result, the range of strong short-range repulsion that originates from hydration and protrusion forces between apposing membranes decreases sharply. This decrease, established more than 3 decades ago by Rand and Parsegian (Rand and Parsegian, 1989), greatly facilitates the close contacts between hydrophobic defects and induces non-linear and monotonic dependencies between the defect density and the nucleation rate. In agreement with this view, it was recently shown that the reduction of the short-range repulsion range induced by surface hydrophobicity induced an exponentially increasing rate of vesicle collisions (Smirnova et al., 2019). This is a critical point showing that the short-range repulsions between the apposing membranes can play a part as important as the energy barriers in the fusion process. In spite of this importance, most simulations of the fusion process currently omit the role of the short-range repulsion, indicating there is little awareness about the “rate” crisis which is reminiscent of the “energy” crisis within elastic continuum models that was resolved 20 years ago (Kozlowsky et al., 2002). Hence we explain the large variation in fusion rate by the presence of hydrophobic defects that, not only change the energy for shape change, but, more critically, also have a profound impact on the short-range repulsion and therefore the short-range collision rate.

A different but related aspect needs to be discussed here. The density of defects is also related to the membrane curvature. Hence, the values established here for the nucleation rate will depend on the curvature. This is similar to what is observed with “curvature-sensing” proteins. Actually, these proteins often sense membrane hydrophobicity rather than curvature (Vamparys et al., 2013; Vanni et al., 2013; Vanni et al., 2014). Hence, depending on the membrane composition they may bind to a highly curved membrane with cylindrical lipids or a flat membrane with conical lipids. The exact same effect is at play here: the more curved the membrane, the higher the hydrophobic defect density and the higher the nucleation rate.

The fact that PDPC membranes show a very low nucleation rate seems in line with our hydrophobic defects hypothesis. Indeed, polyunsaturated lipids are very flexible and should thus freely reorganize to reduce the water/hydrophobic chains contacts. We can suppose there are very few defects and that it is why ν_0_ is so low.

### Optimizing Fusion *In Vivo* Requires Both The Presence Of Conical Shape Lipids And Malleable Lipids

Since fusion-specialized organelles as synaptic vesicles and sperm cells have specific lipid compositions and since lipids seem to be able to impact fusion propensity both through the activation energy and the nucleation rate, we wondered if this double control could play a role *in vivo*. It is noteworthy that synaptic vesicles and sperm cells exhibit a large proportion of polyunsaturated lipids (Pinot et al., 2014) which, according to our predictions, facilitate the processing of fusion from an energy point of view. However, these lipids have a very low nucleation rate, which should not help the organelle to fuse easily. From our conclusions, we hypothesize that lipid malleability may facilitate fusion but requires other lipids to lead to optimum fusion speed and, actually, synaptic also contain a quite large fraction of PE lipids, which is in line with the requirement stated above.

We therefore decided to study a more physiological fusion event and mimicked the fusion that takes place between a synaptic vesicle and the plasma membrane of a neuron during exocytosis of neurotransmitters. We measured the activation energy of the fusion taking place between synaptic vesicle-type vesicles (SV), based on the lipid composition of rat synaptic vesicles (Takamori et al., 2006) and plasma membrane-type vesicles (PM), relying on average plasma membranes (van Meer et al., 2008).

**FIGURE 5:**
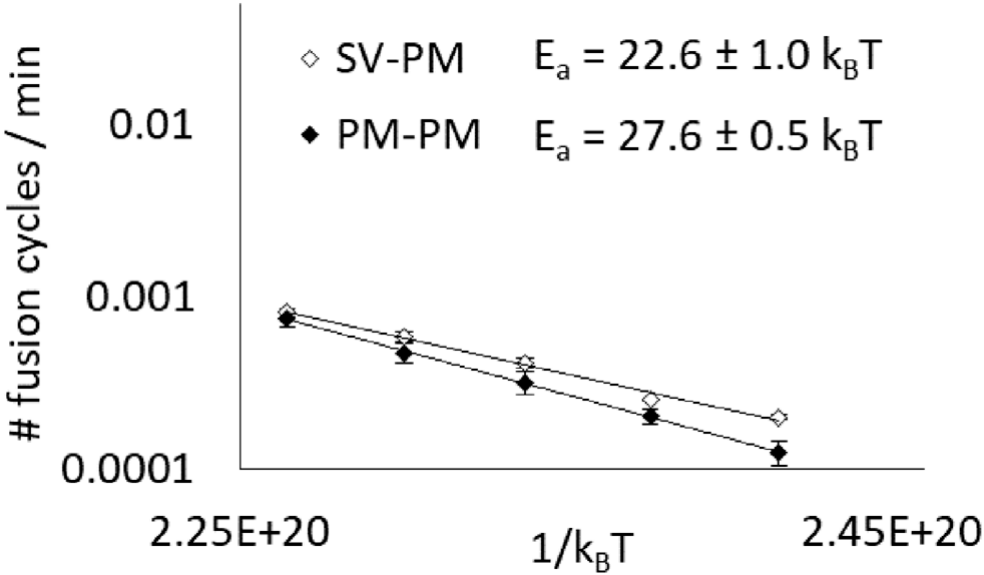
Synaptic vesicle’s composition has a lower fusion activation energy than that of a typical membrane. Average initial spontaneous fusion speeds (in cycles of fusion per minute, i.e. corresponding to the fraction of fusing vesicles per minute) are represented in function of the temperature (from 27 to 47°C) for asymetrical fusion events between PM and SV-type membranes and for PM-PM events, as a control. Activation energy values are determined thanks to independent fits of the different experiments even though, for the sake of clarity, the average fusion speeds are represented (error bars are standard errors on the mean).

We measured an activation energy of 22.6 ± 1.0 k_B_T (Figure 5). To be able to discuss this value and infer on synaptic vesicles’ fusion ability, we compared this value to the activation energy of the fusion between two plasma membrane-type vesicles, which is 27.6 ± 0.5 k_B_T. Our measurements show that synaptic vesicle compositions present a lower fusion activation energy than that of typical plasma membrane compositions, which could potentially help these organelles to fuse easily with the neurons’ plasma membranes to release the neurotransmitters in the synaptic cleft. Nevertheless, it must still be noted that, fortunately, the activation energy is still too high to allow extended spontaneous fusion.

We propose that this could have an importance *in vivo*, where it could allow an easy but tightly regulated membrane fusion, by having a less energy-demanding fusion course than other membranes but requiring external actors to create defects and nucleate fusion when and where it is necessary—let’s say proteins by disturbing the membranes thanks to their *trans*-membrane anchoring.

Lipid composition could thus be a good parameter that biological membranes adjust *in vivo* to regulate their fusion. The presence of two levers could allow to facilitate fusion without enabling it to take place in an anarchic way, by requiring a destabilizing step that could be conducted by proteins.

## Materials and Methods

### Materials

DOPC (1,2-di-(9Z-octadecenoyl)-sn-glycero-3-phosphocholine),POPC (1-palmitoyl-2-oleoyl-sn-glycero-3-phosphocholine), DOPE (1,2-dioleoyl-sn-glycero-3-phosphoethanolamine), PDPC (1-palmitoyl-2-docosahexaenoyl-sn-glycero-3-phosphocholine), NBD-DOPE (1,2-dioleoyl-sn-glycero-3-phosphoethanolamine-N-(7-nitro-2-1,3-benzoxadiazol-4-yl) and Rho-DOPE (1,2-dioleoyl-sn-glycero-3-phosphoethanolamine-N-(lissamine rhodamine B sulfonyl), DPPC (1,2-dipalmitoyl-sn-glycero-3-phosphocholine), 18:0-22:6 PE (1-stearoyl-2-docosahexaenoyl-sn-glycero-3-phosphoethanolamine), 18:0-20:4 PE (1-stearoyl-2-arachidonoyl-sn-glycero-3-phosphoethanolamine), 18(Pl)-22:6 PE (1-(1Z-octadecenyl)-2-docosahexaenoyl-sn-glycero-3-phosphoethanolamine), 18(Pl)-20:4 PE (1-(1Z-octadecenyl)-2-arachidonoyl-sn-glycero-3-phosphoethanolamine), 18:0-22:6 PS (1-stearoyl-2-docosahexaenoyl-sn-glycero-3-phospho-L-serine), SM (N-stearoyl-D-erythro-sphingosylphosphorylcholine), Chol (cholesterol), BrainPC (Sphingomyelin extracted from porcine brain), BrainPE (L-α-phosphatidylethanolamine from porcine brain), BrainPIP2 (L-α-phosphatidylinositol-4,5-bisphosphate from porcine brain), BrainPS (L-α-phosphatidylserine from porcine brain), BrainSM (Sphingomyelin from porcine brain) were purchased from Avanti Polar Lipids and stocked in chloroform and under argon at −20°C. HEPES, KCl and Triton (Triton™ X-100) were purchased from Sigma Aldrich. A ready to use solution (Triton 4% vol/vol) was prepared by diluting 40 μL of Triton in 1 ml of pure water.

### Alexa 647-NHS (Alexa Fluor™ 647 NHS Ester (Succinimidyl Ester)) Was Purchased From ThermoFisher

#### Lipid Vesicles Formation

Lipids are mixed and dried at the bottom of a glass tube. The solvent is evaporated under a flow of nitrogen before staying under vacuum for at least 2 hours. The lipid film is then rehydrated in an HEPES 25 mM—KCl 100 mM buffer at pH = 7.4 to form multilamellar vesicles at 18 mM lipid and vigorously vortexed. The solution is then frozen with liquid nitrogen and thawed 5 times before being extruded 21 times through a 50 nm pore polycarbonate membrane (Avanti Polar Lipids) and let to rest overnight at 4°C for stabilization.

Lipid composition of model plasma membrane vesicles (PM) were determined from the polar head study from Gerrit et al.’s (van Meer et al., 2008). The composition of the model synaptic vesicles (SV) was determined according to the findings of Takamori et al. (Takamori et al., 2006). SV vesicles contained 33% of Chol, 16% of POPC, 5.5% of DPPC, 11% of 18:0–22:6 PE, 5.5% 18:0–20:4 PE, 9% 18(Pl)–22:6 PE, 4.5% 18(Pl)–20:4 PE, 8% 18:0–22:6 PS, 4.5% SM, 1.5% NBD-DOPE and 1.5% Rho-DOPE. PM vesicles contained 50% Chol, 20% BrainPC, 12% BrainPE, 2% BrainPIP2, 5% BrainPS, and 11% BrainSM.

### Lipid Mixing Assay

Fluorescent intensities are measured from the bottom in 96-well clear plates (#353072, Falcon) with a 96-well plate reader (SpectraMax M5e, Molecular Devices). NBD is excited at 460 nm and its emission is read at 538 nm. For the lipid mixing assays, non-labeled liposomes are mixed with fluorescent liposomes.

### Data Treatment

The NBD signal is normalized by its maximum dequenching by adding detergent (Triton 0.66% vol/vol) at the end of the assay. Since the different experiments are done at different temperatures, care should be taken to convert the fraction of NBD dequenching in comparable speeds of fusion (Parlati et al., 1999; François-Martin and Pincet, 2017). The initial speeds of fusion were deduced from the initial slopes of the fusion curves (representing the fraction of already fused vesicles per minute). They are plotted in function of the temperature and fitted by an exponential curve to determine two parameters: the activation energy and the nucleation rate.

### Molecular Dynamics Simulations

Molecular dynamics (MD) simulations were performed with GROMACS 2018.5 (Abraham et al., 2015). The CHARMM36 force field (Klauda et al., 2010) was used for all simulations. Each system was constructed with the CHARMM-GUI webserver (Jo et al., 2008). It consisted of 256 lipids surrounded by 10,240 water molecules (40 waters per lipid) and KCl ions at 100 mM. Four systems were considered made of DOPC/DOPE mixtures at various ratios, namely in terms of number of lipids 256:0, 224:32, 192:64, and 128:128, which translate to 0, 12.5, 25 and 50% of DOPE respectively. The systems were equilibrated using the CHARMM-GUI protocol, that is, an energy minimization followed by several short MD simulations with position restraints on lipids that are progressively released. Then, a MD production run of 500 ns in the NPT ensemble was performed. Electrostatic interactions were calculated with the particle-mesh-Ewald (PME) method (Darden et al., 1993; Essmann et al., 1995), with a real-space cutoff of 1 nm. Van der Waals interactions were computed using a Lennard-Jones force-switching function over 10–12 Å. Bond lengths were constrained using the LINCS algorithm (Hess et al., 1997). The integration time step was set to 2 fs. Water molecules were kept rigid with the SETTLE algorithm (Miyamoto and Kollman, 1992). The system was coupled to a Bussi thermostat (Bussi et al., 2007) and to a semi-isotropic Parrinello–Rahman barostat (Parrinello and Rahman, 1981) at a temperature of 303.15 K and a pressure of 1 atm. MD frames were saved every 100 ps for further analysis.

MD simulations were analyzed in terms of lipid packing defects using PackMem (Gautier et al., 2018). Briefly, packing defects are small hydrophobic patches which are vertically accessible from the outside of the membrane. PackMem evaluates the area of each packing defect from a given MD frame. Then statistics can be accumulated from the many frames of each MD trajectory so that we can extract a distribution of defects which depends on lipid composition and curvature. Note that we used flat membranes in this work. Usually, packing defects are classified as “deep” defects when the hydrophobic patch is at least 1 Å below the central glycerol carbon, or “shallow” defects otherwise. Both deep and shallow defects can be merged into another category that is called “all” defects. It is this latter category that has been used in this study. Rather than characterizing the distribution for each lipid composition as is usually done (Vamparys et al., 2013; Gautier et al., 2018), we simply used the fraction of membrane area occupied by defects. In this work, we have decided to call these lipid packing defects “hydrophobic defects”. Molecular graphics were rendered using VMD (Humphrey et al., 1996).

### DOPE Labelling and Quantification

DOPC liposomes containing a various amount of DOPE (0, 25 and 50%) are prepared as explained above. 1.5% of NBD lipids are included in the lipid mixture to be able to compare samples which do not necessarily have the same lipid concentration. The vesicle solutions are then diluted in buffer to reach a lipid concentration of 1 mM. First, the external DOPE lipids are labeled by adding 100 µg of Alexa 647-NHS to 50 µL of these liposomes. The solution is incubated for 4 h at room temperature under agitation. Then, to get rid of the unbound dyes, the solution is dialyzed over night against 4 L Buffer (cutoff 10,000 kD). The quantification of the DOPE is done thanks to the measurement of the fluorescence intensity $I_{Alexaext}$ of the remaining Alexa 647-NHS (excitation at 647 nm and emission at 671 nm). The liposomes are solubilized through detergent action (Triton at 0.67% v/v), to avoid any fluorescence quenching effect. $I_{Alexaext}$ is normalized by $I_{NBD1}$ , the fluorescence intensity of NBD, that is linked to the total lipid concentration. Then, the remaining unlabeled DOPE (*i.e.* that was initially present on the inner leaflets of the liposomes) are labeled by incubating the sample with another 100 µg of Alexa 647-NHS for 4 h at room temperature under agitation. The solution is dialyzed again over night against 4 L Buffer (cutoff 10,000 kD), leading to a novel formation of liposomes and a very important extraction of the unbound dyes. The total DOPE of the samples is quantified by measuring Alexa’s fluorescence intensity ( $I_{Alexatot}$ ), which is again normalized by NBD intensity ( $I_{NBD2}$ ), to be able to calculate the external fraction of DOPE, that is given through: IAlexaextINBD1IAlexatotINBD2 .

## Data Availability

The original contributions presented in the study are included in the article/Supplementary Material, further inquiries can be directed to the corresponding author.
